# A neural network analysis of Lifeways cross-generation imputed data

**DOI:** 10.1186/s13104-018-4013-2

**Published:** 2018-12-14

**Authors:** Gabrielle E. Kelly

**Affiliations:** School of Mathematics and Statistics, University College Dublin, Belfield, Dublin 4, Dublin, Ireland

**Keywords:** Body mass index, Child, Neural networks, Multiple imputation, Multiple imputation by chained equations, Principal components, Reduction method, Lifeways

## Abstract

**Objectives:**

Neural networks are a powerful statistical tool that use nonlinear regression type models to obtain predictions. Their use in the Lifeways cross-generation study that examined body mass index (BMI) of children, among other measures, is explored here. Our aim is to predict the BMI of children from that of their parents and maternal and paternal grandparents. For comparison purposes, linear models will also be used for prediction. A complicating factor is the large amount of missing data. The missing data may be imputed and we examine the effects of different imputation methods on prediction. An analysis using neural networks (and also linear models) that uses all available data without imputation is also carried out, and is the gold standard by which the analyses with imputed data sets are compared.

**Results:**

Neural network models performed better than linear models and can be used as a data analytic tool to detect nonlinear and interaction effects. Using neural networks the BMI of a child can be predicted from family members to within roughly 2.84 units. Results between the imputation methods were similar in terms of mean squared error, as were results based on imputed data compared to un-imputed data.

## Introduction

Increasing levels of body mass index (BMI) present a problem, particularly in children. Determining possible pathways of familial transmission is important, both for prevention and management intervention. In the Lifeways cross-generation cohort study, expectant mothers were recruited initially in 2001–2003 [1]. Height and weight at age 5 were recorded for 567 children. Where available from either baseline or follow-up, BMI measurements of their parents, maternal grandmother (mgm) and grandfather (mgf), paternal grandmother (pgm) and grandfather (pgf) were also recorded. The extent to which the BMI of the child can be predicted from their parents, maternal and paternal grandparents is of interest. Neural networks (NN) have emerged as a major field of statistics and data analysis where the goal is to create reliable and flexible predictive models. They are described in many books, for example Ripley [2].

As is a common problem in epidemiological studies the Lifeways study has missing data. Multiple imputation (MI) was developed to address the limitations of a complete case analysis [3]. Different imputation models are used here and both NN’s and linear models are fitted to the imputed data. Results are compared to a reduction method where no imputations are done, but predictions are made based on complete data models for the different patterns of missing data.

## Main text

### Methods

#### Neural networks

Neural networks are fitted to these data in order to predict child BMI from (possibly) non-linear functions of the covariates and their interactions. This is not easily done using a linear model, as the number of possible non-linear and higher-order interaction terms is large. We experiment with fitting multi-layer neural networks using the backpropagation algorithm of [4] in which one fits the unknown weight parameters in a neuron using a gradient search method. The networks we use had one or more layers of sigmoidal units with a single sigmoidal output layer. The choice of how many neurons and how many layers is determined by leave-one-out cross validation i.e. one observation is left out at a time and predicted by a NN fit to the remainder of the data. The error-difference between actual and predicted is computed, squared and the average taken over all observations. This will be referred to as mean squared error (mse). The network that minimizes this error is chosen. MSE is also used as a criterion to assess goodness-of-fit of the model. Different starting weights may give different NN’s and thus different mse. Therefore we fit each NN with 3 repetitions and the minimum error for each observation is taken. Non-linearity in the model is also explored using a generalized additive model (GAM) in which the linear predictor is given by a sum of smooth functions of the covariates [5]. It’s main difference from a NN model is that interaction terms are not automatically included. It is known GAM models underestimate p-values and they are not our main interest.

#### Multiple imputation

A complete case analysis of these data would involve very few families (< 15) and statistically more powerful analyses can be done by including individuals with incomplete data. The MI methods used to do this require the missing at random assumption to produce unbiased estimates [6]. An investigation into the pattern of missing data in [7] did not demonstrate any systematic variability and these data were found to be missing completely at random.

MI is a two-stage process. In stage 1, the incomplete data set is replicated multiple times, and missing values are replaced by plausible values drawn from a posterior distribution according to a suitable imputation model based on the observed data. In stage 2, the target analysis is performed on each of the imputed data sets with the resulting parameter estimate and corresponding standard error of each data set, combined into a single estimate (and standard error) using Rubin’s rules [3]. For stage 1, we use an imputation model based on principal component analysis (PCA) due to [8] that is fit using their R package missMDA. The first step consists in imputing the missing entry with an initial value, then PCA is performed on the imputed data set. Then, the value fitted by PCA is used to predict a new value for the missing one. On the new completed data set, the same procedure is applied and these two steps are repeated until convergence. To prevent over-fitting when there are many missing values a regularized version of the algorithm is available.

We also use a fully conditional specification (FCS), also known as multiple imputation by chained equations (MICE), that fits separate univariate models to each variable with missing values, iteratively cycling through the univariate models. Univariate imputation models considered here are Bayesian linear regression (NORM) and predictive mean matching (PMM). For NORM as described in [9], we assume that z is a variable whose missing values we wish to impute from other (complete) variables $x=(x_1, \ldots , x_k ),$ including an intercept term. Let *nz* be the number of individuals with observed z values. We assume $z|x;\beta \sim N (\beta x,\sigma ^2)$. Let $\hat{\beta }$ and V be the set of estimated regression parameters and corresponding covariance matrix from fitting this model. Let $\beta ^*$ be a random draw from the posterior distribution given by $\beta ^* \sim MVN (\hat{\beta },V)$. Imputations for *z* are drawn from the posterior predictive distribution of *z* using $\beta ^*$.

PMM reduces the impact of model mis-specification, or non-normality [9]. In PMM, using a perturbed parameter vector $\beta ^*$ as above, for each missing value $z_i$ with covariates $x_i,$ the q individuals with the smallest values of $|\hat{\beta }x_h-\beta ^*x_i|$ $(h=1, \ldots ,nz)$ are identified. One of these q closest individuals, say $i^{\prime }$, is chosen at random, and the imputed value of $z_i$ is $z_{i^{\prime }}$. We use $\hbox {q}=3$, which performed well in a simulation study [9].

The R package mice [10] is used to carry out these procedures. There is no mechanism at present that allows imputation to be done using a NN model, as a NN model is not identifiable.

#### Gold standard-network reduction method

All patterns of missing data were identified and a NN and a linear model fit to each data set with those variables that are present [11].

#### Statistical analysis

Summary statistics for these data including an analysis involving pairwise correlations and univariate linear regression models may be seen in [7].

The imputation methods PCA, PMM and NORM are carried out on the data and 10 imputed data sets obtained for each method. Each imputed data set is fit using a NN and a linear model.

Neural net fitting is done using two layers, the first with two neurons and the second with one, as this provided close to the best fit in terms of mse and had a low computation time. This is done using the neuralnet package in R [12]. As computing leave-one-out mse involves 567 NN fits, and 3 replications for each fit are carried out, the number of data sets imputed for each imputation method was set to ten. The linear models regress child BMI on a linear combination of family member’s BMI.

For the reduction method, since the data set contain 63 patterns of missing values, 63 NN and also 63 linear models are fitted. To provide comparison with other methods, each NN is trained on 80% of the available data and the test set is the remaining 20%. The mse is computed and then the average mse over the 63 NN’s computed. A similar procedure is conducted for the linear model. Computations are carried out in R [13].

The GAM model is fit using the mgcv package in R with the default options [14].

### Results

The fraction of missing data for each family member is child = 0.0, mother = 0.012, father = 0.579, mgm = 0.561, mgf = 0.716, pgm = 0.679 and pgf = 0.774. The root mse in the NN models is roughly 1.45 units of BMI. Thus the BMI of a child can be predicted from family members to within $\pm \,1.96 \times 1.45 = \pm \,2.84$. The following can be inferred from Tables 1 and 2:

- MSE is smallest on data imputed using MICE and PMM, followed by MICE with Bayesian linear regression and worst for data imputed with PCA. This is also true for the linear models fit.
- The neural net gave a smaller mse than the linear model for each imputation method indicating some non-linearity and interaction terms in the linear model.
- Imputing with MICE and PMM and fitting with a neural net gave a slightly smaller mse than the reduction method fit with neural net but the reverse is true for linear models. The standard deviation of mse for the reduction method linear models (63 in all) is large. This is as expected as some of the data sets fitted are quite small e.g. data sets with 6 or 7 family members.
- The important predictors in the linear model ($p < 0.05$) are mother, father and mgm for all imputation methods, except PCA where the mgm is borderline significant.

It is not possible to identify the important predictors for a NN with any data or the linear model reduction method. For a NN the weights for any particular neuron are not identifiable. The linear model reduction method involves 63 linear models, each with different predictors, with no obvious way of combining the results. The $R^2$ value was available for each model via the mse.

**Table 1:** Estimates (s.e.) and p-values for each family member in a linear model

Member	Imputation by PCA*			Imputation by MICE and PMM			Imputation by MICE and Norm		
	Estimate	s.e.	p-value	Estimate	s.e.	p-value	Estimate	s.e.	p-value
Mother	0.0614	0.0178	0.0006	0.0519	0.0191	0.0065	0.0549	0.0190	0.0038
Father	0.0563	0.0232	0.0155	0.0900	0.0263	0.0006	0.0716	0.0355	0.0438
Mgm	0.0325	0.0183	0.0757	0.0493	0.0216	0.0224	0.0441	0.0157	0.0049
Mgf	0.0413	0.0267	0.1212	0.0379	0.0325	0.2430	0.0275	0.0383	0.4703
Pgm	0.0142	0.0247	0.5635	0.0073	0.0192	0.7023	0.0074	0.0205	0.7171
Pgf	0.0094	0.0202	0.6424	− 0.0028	0.0528	0.9582	− 0.0155	0.0324	0.6327

* Using prediction rules for the imputed data sets

**Table 2:** Leave-one-out mse (sd) for different models for the 10 imputed and the reduction data sets

Imputation method	Prediction rule mse	Mse sd	$R^2$
Neural network			
PCA	2.3271	0.0854	22.23
MICE and PMM	2.1106	0.1181	29.46
MICE and NORM	2.2938	0.0965	23.34
Reduction method	2.1662	1.0207	27.60
Linear model			
PCA	2.7083	0.0467	9.50
MICE and PMM	2.5674	0.0980	14.20
MICE and NORM	2.6367	0.0780	11.88
Reduction method	2.3033	0.9625	23.02

$R^2$ is the percentage of variation explained

**Fig. 1:**
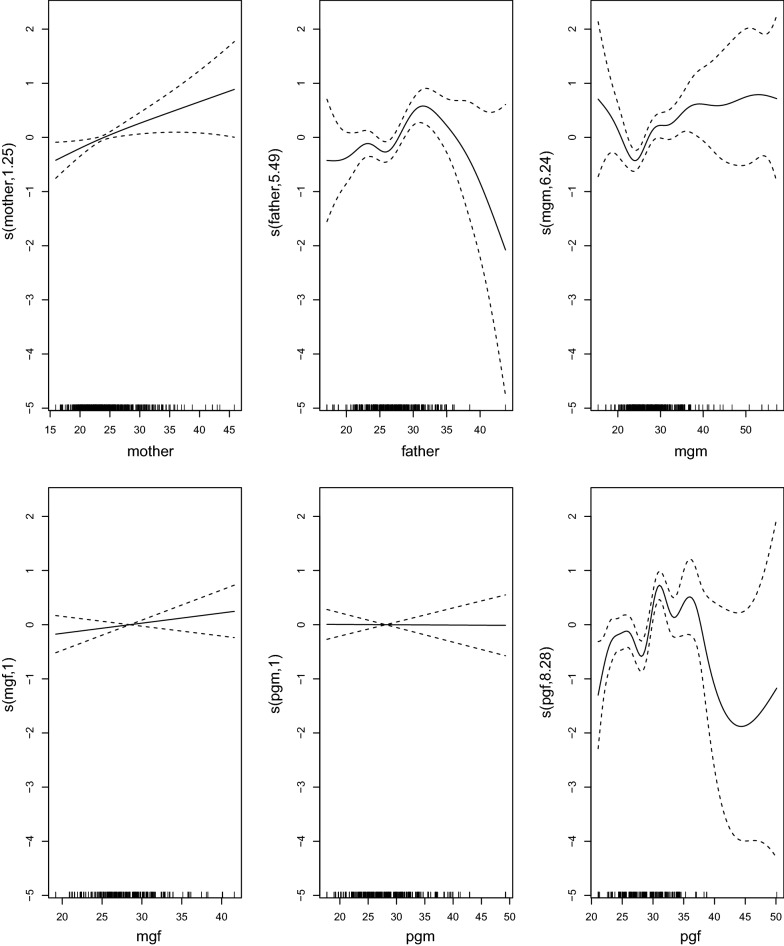
Typical relationships between child and family members using a GAM model. The figure shows the component smooth functions for each covariate that make up the GAM fit, on the scale of the linear predictor, with associated confidence bands

The results of the GAM fit to one data set imputed by PMM are shown in Fig. 1. This shows clearly non-linearity in the response of the child with father, mgm and pgf. We also note, results not shown, that mse for GAM models in Table 2 are between those for linear models and NN’s, as is expected. The $R^2$ for a GAM model fitted to data imputed by MICE and PMM was 19.70%, considerably less than the neural net value in Table 2. This indicates interaction terms (not automatically included in GAM models) are important in the prediction.

### Discussion

NN’s give the best predictions of the methods considered. They also provide a measure of the degree of non-linear and interaction effects in the data that can aid in identifying suitable models.

The advantages of this study is that it uses data from three generations of one family. To our knowledge no other study has attempted to predict child BMI from the two previous generations.

In [7] univariate linear regression models were conducted and a maximum $R^2$ of 4% was observed between child and mother. That between child and mgm was also 4%. However one of the main aims of that study was to model associations between family members and to understand the underlying process generating the data. Here the aim is to establish how well child BMI can be predicted from that of family members.

De Silva et al. [15] evaluated the performance of different MI methods including NORM for handling up to 50% of missing data when assessing the association between childhood obesity and sleep problems. They also conducted a simulation study and observed slight gains in precision for all MI methods when compared with a complete case analysis. Our results are in agreement with this in terms of comparing imputed data models with a high percentage of missing data with reduction methods. There is little difference between analyses conducted on imputed data and analyses conducted on reduced data—where no imputation is done.

The network reduction method is computationally intensive to implement but its use as a gold standard to compare imputed methods is a useful technique in studies such as this, where large amounts of data are imputed.

Our results represent valuable information regarding protocol and data collection in relation to this and similar studies. They indicate that studies based on incomplete data where missing data is imputed can give reliable results.

## Limitations

- The NN architecture used is relatively simple. Slightly better results can be obtained for more complicated NN’s as exploratory analyses revealed.
- There is no mathematical theory to justify MICE and PCA imputation methods in general and no simulation can study all the possibilities. We used real data to assess deviations from observations and results are limited to these data.

## Abbreviations

BMI: body mass index
Mgm: maternal grandmother
Mgf: maternal grandfather
Pgm: paternal grandmother
Pgf: paternal grandfather
NN: neural network
MI: multiple imputation
Mse: mean squared error
GAM: generalized additive model
PCA: principal components analysis
FCS: fully conditional specification
MICE: multiple imputation by chained equations
NORM: univariate imputation by Bayesian linear regression
PMM: predictive mean matching
